# Differences in Trauma Injury Patterns and Severity Between Intentional and Accidental Falls From a Height: A Japanese Nationwide Trauma Database Study

**DOI:** 10.7759/cureus.25861

**Published:** 2022-06-12

**Authors:** Takero Terayama, Hiroyuki Toda, Yoshihiro Tanaka, Daizo Saitoh, Aihide Yoshino

**Affiliations:** 1 Traumatology and Critical Care Medicine, National Defense Medical College Hospital, Tokorozawa, JPN; 2 Psychiatry, School of Medicine, National Defense Medical College, Tokorozawa, JPN; 3 Traumatology and Critical Care Medicine, National Defense Medical College, Tokorozawa, JPN

**Keywords:** injury patterns, trauma database, jumping, suicide, fall from a height

## Abstract

Background

Fall from a height is a common cause of trauma requiring emergency care; in many cases, the trauma team needs to urgently develop the initial treatment strategy. The mechanism of injury (intentional or accidental) is an important factor in predicting trauma patterns and severity. We aimed to describe how the severity of injuries in each body region contributes to overall trauma severity and skeletal trauma patterns in intentional and accidental falls.

Methods

Data accumulated between January 1, 2004 and May 31, 2019 were obtained from a nationwide trauma database. Patients aged ≥18 years and injured by falls from a height were included. The median Abbreviated Injury Scale (AIS) score for the Injury Severity Score (ISS) for each body region (region 1: head, face, and neck; region 2: thorax; region 3: abdomen; region 4: lower extremity and pelvis; and region 5: upper extremity) was investigated. Skeletal injury patterns were classified into four groups: group I (intentional/severe), group II (accidental/severe), group III (intentional/not severe), and group IV (accidental/not severe). Severe trauma was defined as a trauma with an ISS of 16 or more. The groups were compared using the chi-square test and Mann-Whitney U test.

Results

Among the 342,263 patients enrolled in the database, 28,409 met the inclusion criteria: 6,812 in group I, 11,754 in group II, 2,384 in group III, and 7,459 in group IV. The intentional fall group showed an increase in the AIS score for region 4 as the ISS increased, whereas the accidental fall group showed an increase in the AIS score for region 1. Both groups showed an increase in the AIS score for region 2 as the ISS increased. The intentional fall group had a higher proportion of fractures in the lower extremities and pelvis than the accidental fall group.

Conclusions

There were differences in trauma patterns and trauma severity levels between patients who experienced intentional and accidental falls from a height. Our findings provide a comprehensive understanding of this topic. Further studies are required to assess the usefulness of our findings for the development of initial treatment strategies at the ED.

## Introduction

Fall from a height is one of the most common causes of severe trauma observed at emergency departments (EDs), and it is associated with high rates of death and significant disability due to the characteristic distribution of bodily injuries [1,2].

At the ED, patients who fall from a height often require collaborative treatment, involving the expertise of physicians from various departments, such as neurosurgeons, orthopedic surgeons, and thoracic surgeons. Thus, predicting injury patterns as soon as possible at the ED helps trauma team leaders develop an appropriate initial treatment strategy. For this purpose, epidemiological data on the distribution of fractures and trauma severity would be useful. Skeletal injury patterns and trauma severity due to falls from a height have been investigated, focusing on the mechanism of injury (intentional or accidental) [2-7]. Previous studies have reported that intentional falls caused more fractures in lower extremities than unintentional falls [2,5,7].

However, these findings should be interpreted with caution owing to the heterogeneity of trauma severity in the intentional and the accidental fall groups in the previous studies, and to our knowledge, no study had reported the differences in both trauma patterns and overall severity using the Abbreviated Injury Scale (AIS) score and Injury Severity Score (ISS). Small sample sizes and single-center case series design are also some of the limitations of previous studies.

Therefore, we aimed to investigate the differences in both skeletal trauma patterns and trauma severity between intentional and accidental falls from a height using a large sample population from a nationwide trauma database.

## Materials and methods

Ethical statement

This study adhered to the principles of the Declaration of Helsinki. This study was approved by the Institutional Review Board of the National Defense Medical College. Informed consent was not required because this was an observational study that included only existing medical information. This study followed the Strengthening the Reporting of Observational Studies in Epidemiology (STROBE) guidelines [8].

Study design

This study was a retrospective data review of a multicenter, nationwide trauma database.

Data source

The Japanese Trauma Databank (JTDB) is a multicenter, nationwide trauma registry in Japan established in 2003 by the Japanese Association for the Surgery of Trauma and by the Japanese Association for Acute Medicine with the aim of improving the quality of trauma care in Japan. The data of patients who were transported to the participating hospitals and had at least one AIS score of 3 or higher were registered in the JTDB. Patients who refused to participate in the JTDB were excluded. As of 2021, 292 acute care hospitals that provide trauma care throughout Japan were participating in the JTDB. The data were entered by physicians and medical assistants who had attended AIS-coding lectures. The data in the JTDB includes approximately 90 items, covering prehospital care, initial treatment, diagnosis, in-hospital treatment, and clinical outcomes such as mortality and length of hospital stay. In this study, we used the JTDB, which was released in 2021, and included trauma patients treated between January 1, 2004 and May 31, 2019.

Participants

Patients who were aged ≥18 years and were injured by intentional and accidental falls from a height were included. We excluded patients with missing ISS data.

Data collection

The data relating to patients and hospital information were obtained from the JTDB, including demographic information, pre-hospital, emergency department, in-hospital treatments, AIS score, ISS, and clinical outcomes.

Definitions of the variables

The AIS is an internationally accepted tool for ranking injury severity. The AIS is an anatomically based, global severity scoring system that classifies each injury by body region according to its relative severity on a six-point scale (one = minor, six = lethal). In this study, the AIS 98 was used [9]. The AIS scores were originally calculated separately for nine regions (head, face, neck, thorax, abdomen, spine, upper extremity, lower extremity, and others, which included external and thermal injuries and other trauma), but we re-grouped these nine regions into five for our analyses: region 1 (head, face, and neck), region 2 (thorax), region 3 (abdomen), region 4 (lower extremity and pelvis), and region 5 (upper extremity). The cervical, thoracic, and lumbar spine/spinal cord regions were classified under regions 1, 2, and 3, respectively. The AIS provides the foundation for the ISS, a recognized tool for assessing overall injury severity. The ISS is the sum of the squares of the highest AIS code in each of the three most severely injured ISS body regions [9,10]. Severe trauma was defined as a trauma with an ISS of 16 or higher. The cut-off score of 16 is used in Japan; for certification of trauma centers and trauma specialists, experienced in treating a certain number of patients with ISS of 16 or higher is required.

The included patients were divided into four groups: group I (intentional/severe), group II (accidental/severe), group III (intentional/not severe), and group IV (accidental/not severe). We also classified groups I and III as the intentional fall group, and groups II and IV as the accidental fall group. Whether the injury was intentional or accidental was identified based on the mechanism of injury (intentional, accidental, assaulted, or unknown) in the JTDB. Fractures were identified by AIS codes registered for each patient.

The primary outcome was the distribution of injury severity. We planned to describe how the severity of injury in each body region contributed to overall trauma severity in the intentional and accidental fall groups. The overall trauma severity was defined by the ISS. We compared the median AIS scores for the ISS of each region among the groups. The secondary outcome was skeletal fracture patterns. We also planned to describe the differences in skeletal fracture patterns and compare the characteristics of non-survivors among the groups.

Statistical analysis

The variables were expressed as medians (interquartile ranges: IQRs) for continuous variables and as numbers (percentages) for categorical variables [8]. The characteristics of the four groups defined above were compared. The differences in the regional scores contributing to overall trauma severity in the intentional and accidental fall groups were presented using a graph that plotted the median AIS scores for the ISS of each region. Continuous variables were compared between intentional and accidental fall groups using the Mann-Whitney U or Kruskal-Wallis test, and categorical variables were compared using the chi-squared test.

All analyses were performed using SPSS (IBM SPSS Statistics for Windows, Version 28.0. IBM Corp, Armonk, NY). Statistical tests were two-sided, with p <0.05 indicating statistical significance.

## Results

Selection of patients

**Figure 1:**
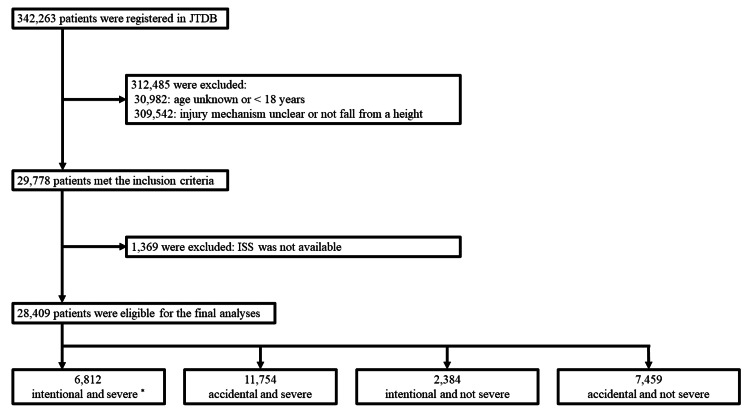
Patient enrolment flowchart JTDB, Japanese Trauma Data Bank; ISS, Injury Severity Score. * ISS ≥ 16 is considered “severe”.

Figure 1 summarizes the process of selecting patients for this study. Among the 342,263 patients registered in the JTDB, 28,409 patients were eligible. Of these, 6,812 (24.0%) were classified under group I (intentional/severe), 11,754 (41.4%) under group II (accidental/severe), 2,384 (8.4%) under group III (intentional/not severe), and 7,459 (26.3%) under group IV (accidental/not severe).

Characteristics of patients

Table 1 demonstrates the characteristics of the patients included in each group. The patients in the intentional fall groups (group I and III) were younger than those in the accidental fall groups (group II and IV). The median ages in groups I, II, III, and IV were 40 years (IQR, 29-55 years), 61 years (44-71 years), 34 years (25-47 years), and 56 years (40-68 years), respectively. The intentional fall groups had lower proportions of men (48.7% in group I, 86.5% in group II, 38.6% in group III, and 85.3% in group IV;* p *< 0.001) and past medical history of psychiatric diseases (45.0% in group I, 5.3% in group II, 60.0% in group III, and 5.0% in group IV; *p* < 0.001) at admission than the accidental fall groups. The mortality rates in the intentional fall groups were twice as high as those in the accidental fall groups. Comparisons between the intentional and accidental fall groups are shown in Table 2; there were statistically significant differences in all variables.

**Table 1:** Characteristics of included patients BP, blood pressure; GCS, Glasgow Coma Scale; ISS, Injury Severity Score; TRISS, Trauma and Injury Severity Score; Ps, Probability of survival; AIS, Abbreviated Injury Scale * ISS ≥ 16 is defined as “severe”. ‡ Including patients with cardiopulmonary arrest on arrival at the emergency department. † AIS score in each region was recalculated for this study from the data registered in the Japanese Trauma Databank.

	Missing data	Group I	Group II	Group III	Group IV	p-value
		Severe^*^		Not Severe		
		Intentional	Accidental	Intentional	Accidental	
N		6,812	11,754	2,384	7,459	
Age, years	0.3%	40 (29–55)	61 (44–71)	34 (25–47)	56 (40–68)	< 0.001
Sex, male	0.02%	3,317 (48.7%)	10,169 (86.5%)	921 (38.6%)	6,358 (85.3%)	< 0.001
Past medical history, Psychiatric disease	0%	3,067 (45.0%)	627 (5.3%)	1,426 (60.0%)	369 (5.0%)	< 0.001
Alcohol use at admission	42.2%	386 (13.7%)	760 (10.5%)	305 (22.6%)	520 (10.3%)	< 0.001
Vital signs at admission‡						
Heart rate, /min	3.3%	82 (0–111)	67 (80–98)	76 (90–104)	69 (79–91)	< 0.001
Systolic BP, mmHg	2.1%	76 (0–114)	100 (128–150)	104 (120–136)	120 (137–155)	< 0.001
Diastolic BP, mmHg	11.9%	58 (0–77)	62 (78–92)	62 (74–86)	70 (81–93)	< 0.001
Respiratory Rate, /min	7.7%	18 (0–25)	16 (20–25)	17 (20–25)	17 (20–24)	< 0.001
Body Temperature, ℃	17.5%	36 (35–37)	36 (36–37)	36 (37–37)	36 (37–37)	< 0.001
GCS score	4.4%	6 (3–14)	14 (9–15)	13 (15–15)	15 (15–15)	< 0.001
Admission to the general ward	0%	269 (4.0%)	1,874 (15.9%)	456 (19.1%)	2,877 (38.6%)	< 0.001
ISS	0%	34 (24–45)	25 (18–33)	9 (8–13)	9 (8–13)	< 0.001
TRISS Ps, %	12%	0.49 (0.02–0.94)	0.89 (0.63–0.94)	0.99 (0.98–0.99)	0.98 (0.96–0.99)	< 0.001
Maximum AIS score in region 1 (head, face, and neck) ^†^	0%	2 (0–4)	3 (0–4)	0 (0–1)	0 (0–2)	< 0.001
Maximum AIS score in region 2 (thorax)	0%	4 (2–5)	3 (0–4)	0 (0–0)	0 (0–2)	< 0.001
Maximum AIS score in region 3 (abdomen)	0%	0 (0–2)	0 (0–2)	0 (0–2)	0 (0–2)	< 0.001
Maximum AIS score in region 4 (lower limbs)	0%	3 (2–4)	0 (0–2)	2 (0–2)	0 (0–2)	< 0.001
Maximum AIS score in region 5 (upper limbs)	0%	0 (0–2)	0 (0–2)	0 (0–0)	0 (0–1)	< 0.001
In-hospital mortality	0%	3266 (47.9%)	2408 (20.5%)	132 (5.5%)	149 (2.0%)	< 0.001

**Table 2:** Comparison of characteristics of patients in the intentional and accidental fall group BP, blood pressure; GCS, Glasgow Coma Scale; ISS, Injury Severity Score; TRISS, Trauma and Injury Severity Score; Ps, Probability of survival; AIS, Abbreviated injury scale. * Including patients with cardiopulmonary arrest on arrival at the emergency department. † The maximum AIS score in each region is obtained from the data registered in the Japanese Trauma Databank.

	Intentional	Accidental	p-value
N	9,196	19,213	
Age, years	39 (28–53)	59 (42–70)	< 0.001
Sex, male	4,238 (46.1%)	16,527 (86.0%)	< 0.001
Past medical history, Psychiatric disease	4,493 (48.9%)	996 (5.2%)	< 0.001
Alcohol use at admission	691 (16.6%)	1,280 (10.4%)	< 0.001
Vital signs at admission^*^			
Heart rate, /min	86 (0–109)	80 (68–95)	< 0.001
Systolic BP, mmHg	97 (0–123)	132 (110–152)	< 0.001
Diastolic BP, mmHg	64 (40–80)	80 (66–92)	< 0.001
Respiratory rate, /min	19 (0–25)	20 (16–24)	< 0.001
Body temperature, ℃	36.2 (35.4–36.8)	36.4 (35.9–36.8)	< 0.001
GCS score	12 (3–15)	15 (13–15)	< 0.001
ISS	26 (14–41)	17 (10–26)	< 0.001
TRISS Ps, %	0.88 (0.054–0.98)	0.95 (0.85–0.98)	< 0.001
Maximum AIS score in region 1 (head, face, and neck) ^†^	1 (0–3)	2 (0–4)	< 0.001
Maximum AIS score in region 2 (thorax)	3 (0–4)	2 (0–3)	< 0.001
Maximum AIS score in region 3 (abdomen)	0 (0–2)	0 (0–2)	< 0.001
Maximum AIS score in region 4 (lower extremity and pelvis)	2 (0–3)	0 (0–2)	< 0.001
Maximum AIS score in region 5 (upper extremity)	0 (0–2)	0 (0–2)	0.003
Death at emergency department	2,515 (27.3%)	1,066 (5.5%)	< 0.001

The characteristics of patients who died are shown in Table 3. The intentional fall group had higher ISS (median, IQR: 41, 27-50 in the intentional fall group vs. 29, 25-41 in the accidental fall group; p < 0.001) and higher maximum AIS scores in region 4 (3, 2-4 vs. 0, 0-2; p < 0.001) than the accidental fall group.

**Table 3:** Comparison of characteristics of non-surviving patients in the intentional and accidental groups BP, blood pressure; GCS, Glasgow Coma Scale; ISS, Injury Severity Score; TRISS, Trauma and Injury Severity Score; Ps, Probability of survival; AIS, Abbreviated Injury Scale * Including that of patients who experienced cardiopulmonary arrest before arrival at the emergency department † The maximum AIS score in each region was obtained from the data registered in the Japanese Trauma Databank.

	Intentional	Accidental	p-value
N	3,398	2,557	
Age, years	45 (32–62)	67 (53–78)	< 0.001
Sex, male	1,855 (54.6%)	2,069 (80.9%)	< 0.001
Past medical history Psychiatric disease	1,049 (30.9%)	165 (6.5%)	< 0.001
Alcohol use at admission	98 (7.9%)	80 (7.7%)	0.81
Vital signs at admission^*^			
Heart rate, /min	0 (0–91)	112 (67–153)	< 0.001
Systolic BP, mmHg	0 (0–50)	67 (37–92)	< 0.001
Diastolic BP, mmHg	0 (0–20)	20 (10–26)	< 0.001
Respiratory rate, /min	0 (0–104)	85 (57–109)	< 0.001
Body temperature, ℃	35.4 (34.3–36.2)	35.9 (35–36.5)	< 0.001
GCS score	3 (3–5.5)	4 (3–10)	< 0.001
ISS	41 (27–50)	29 (25–41)	< 0.001
TRISS Ps, %	0.054 (0.009–0.31)	0.34 (0.074–0.69)	< 0.001
Maximum AIS score in region 1 (head, face, and neck) ^†^	3 (0–4)	4 (3–5)	< 0.001
Maximum AIS score in region 2 (thorax)	4 (3–5)	3 (0–4)	< 0.001
maximum AIS score in region 3 (abdomen)	0 (0–2)	0 (0–2)	0.65
Maximum AIS score in region 4 (lower extremity and pelvis)	3 (2–4)	0 (0–2)	< 0.001
Maximum AIS score in region 5 (upper extremity)	0 (0–2)	0 (0–1)	< 0.001

Distribution of trauma severity in intentional and accidental falls from a height

**Figure 2:**
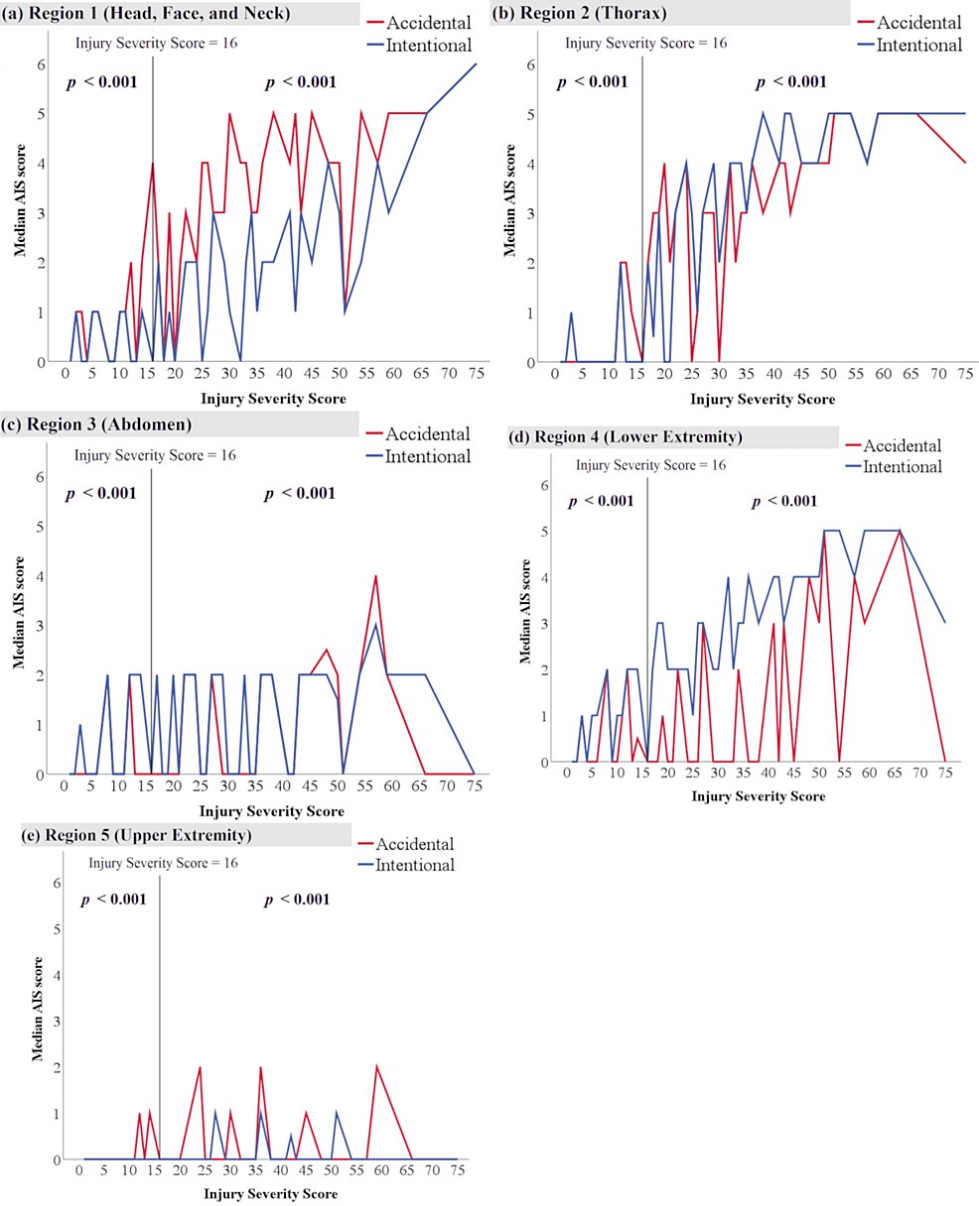
Comparison of the median AIS score for the ISS of each region in the intentional and accidental fall groups AIS, Abbreviated Injury Scale The median AIS score in each region: (a) region 1 (head, face, and neck), (b) region 2 (thorax), (c) region 3 (abdomen), (d) region 4 (lower extremities and pelvis), and (e) region 5 (upper extremities) Comparison between group III (intentional and not severe) and group IV (accidental and not severe). † Comparison between group I (intentional and severe) and group II (accidental and severe).

Figure 2 shows the median AIS scores for the ISS of each region in the intentional and accidental fall groups. In region 1 (head, face, and neck), the median AIS score at the ISS of 75 in both groups was six. As the ISS increased, the median AIS score in the accidental fall group increased earlier than that of the intentional fall group (Figure 2a). There were statistically significant differences in the AIS score between groups I and II and between groups III and IV (p < 0.001). In region 2 (thorax), as the ISS increased, the median AIS score increased in a similar fashion in both the intentional and accidental fall groups. For patients with an ISS between 35 and 50, the median AIS score in the intentional fall group was higher than that in the accidental fall group (Figure 2b). In the case of patients with an ISS below 45 for region 3 (abdomen), the median AIS score was higher in the intentional fall group than in the accidental fall group, and vice versa in the case of patients with an ISS above 45, although the median AIS score in both groups was not higher than three (Figure 2c). For region 4 (lower extremity and pelvis), the median AIS score in the intentional fall group was higher than that in the accidental fall group, regardless of the ISS. In particular, the median AIS score in the intentional fall group was often higher than 4 in patients with an ISS above 30 (Figure 2d). There was a statistically significant difference in the AIS score for region 5 between the groups, but the median AIS score in both groups was below two (Figure 2e).

Skeletal trauma patterns in the four groups

Table 4 summarizes the skeletal trauma patterns in the four groups and the proportion of patients with fractures for each bone. The severe groups (group I and II) had high proportions of the skull (24.8% in group I and 26.4% in group II), rib (50.1% and 46.3%), lumbar spine (29.5% and 22.8%), and pelvis fractures (60.2% and 25.4%). Similarly, the non-severe groups (group III and IV) had high proportions of rib (9.3% in group III and 19.2% in group IV), lumbar spine (35.3% and 20.0%), and pelvis fractures (24.8% and 15.8%).

**Table 4:** Skeletal trauma patterns in the groups * Injury Severity Score ≥16 was defined as “severe”.

	Group I	Group II	Group III	Group IV	p- value
	Severe^*^		Not Severe		
	Intentional	Accidental	Intentional	Accidental	
N	6,812	11,754	2,384	7,459	
Skull	1,688 (24.8%)	3,104 (26.4%)	90 (3.8%)	426 (5.7%)	< 0.001
Facial bone	1,144 (16.8%)	1,199 (10.2%)	198 (8.3%)	427 (5.7%)	< 0.001
Cervical spine	789 (11.6%)	1,199 (10.2%)	70 (2.9%)	470 (6.3%)	< 0.001
Rib	3,415 (50.1%)	5,447 (46.3%)	221 (9.3%)	1,429 (19.2%)	< 0.001
Sternal	261 (3.8%)	340 (2.9%)	21 (0.9%)	105 (1.4%)	< 0.001
Thoracic spine	1,096 (16.1%)	1,847 (15.7%)	236 (9.9%)	844 (11.3%)	< 0.001
Lumbar spine	2,007 (29.5%)	2,677 (22.8%)	842 (35.3%)	1,493 (20.0%)	< 0.001
Scapula	463 (6.8%)	961 (8.2%)	24 (1.0%)	239 (3.2%)	< 0.001
Humerus	869 (12.8%)	454 (3.9%)	127 (5.3%)	253 (3.4%)	< 0.001
Radius or ulna	869 (12.8%)	454 (3.9%)	127 (5.3%)	253 (3.4%)	< 0.001
Hand	119 (1.8%)	251 (2.1%)	48 (2.0%)	198 (2.7%)	0.002
Pelvis	4,101 (60.2%)	2,987 (25.4%)	591 (24.8%)	1,179 (15.8%)	< 0.001
Femur	1,775 (26.1%)	1,105 (9.4%)	290 (12.2%)	811 (10.9%)	< 0.001
Patella	190 (2.8%)	180 (1.5%)	79 (3.3%)	140 (1.9%)	< 0.001
Tibia	879 (12.9%)	472 (4.0%)	272 (11.4%)	534 (7.2%)	< 0.001
Fibula	538 (7.9%)	297 (2.5%)	154 (6.5%)	382 (5.1%)	< 0.001
Foot	287 (4.2%)	143 (1.2%)	186 (7.8%)	129 (1.7%)	< 0.001
Calcaneus	788 (11.6%)	342 (2.9%)	445 (18.7%)	498 (6.7%)	< 0.001
Talus	128 (1.9%)	59 (0.5%)	55 (2.3%)	58 (0.8%)	< 0.001

There was a distinct difference in lower extremity and pelvis fractures between the intentional and accidental fall groups. The intentional fall group had higher proportions of fractures of the lower extremities and pelvis, and the difference was more pronounced in the severe group. The proportions of pelvis fractures were 60.2% in group I, 25.4% in group II, 24.8% in group III, and 15.8% in group IV (p < 0.001). The proportions of calcaneus fractures were 11.6% in group I, 2.9% in group II, 18.7% in group III, and 6.7% in group IV (p < 0.001).

## Discussion

In this study, we demonstrated the differences in the trauma patterns and distribution of trauma severity in patients who experienced intentional and accidental falls from a height, using a large sample population from a nationwide trauma database. The data were classified into four groups according to injury mechanism (intentional or accidental) and trauma severity (ISS ≥ 16 or not).

The most important findings of our results were as follows: First, in the intentional fall groups, the trauma severity increased in the lower extremities and pelvic region as the ISS increased, while in the accidental fall groups, the trauma severity in the head region increased. Second, both the intentional and accidental fall groups showed an increase in thoracic trauma severity as the ISS increased, although this pattern was observed sooner in the accidental fall group. Last, the intentional fall group had more fractures of the lower extremity and pelvis than the accidental fall group.

These findings would help emergency physicians to develop an initial treatment strategy for trauma patients injured by falls from a height at the ED. For example, critically severe patients may require surgery over the head, chest, and pelvic regions. On the other hand, if not, patients who attempt intentional falls may be more likely to require interventional radiology or other therapeutic intervention to the pelvis than those who are injured by non-suicidal falls. Some patterns may be predictable at the phase of the decision to receive patients from emergency medical services.

Several studies have reported partially similar findings to our results [2,4-7]. For example, Papadakis et al. reported the distribution of fractures in the pelvis (42.1% in the intentional fall group vs. 4.4% in the accidental fall group, p < 0.001), ribs (40.6% vs. 25.5%, p < 0.001), lower extremities (1.1%-9.4% vs. 14%-59.3%), and skull (25% vs. 3.8%, p < 0.001) [2]. Our study showed that the intentional fall group comprised a higher proportion of patients with skull fractures than the accidental fall group. Faggiani et al. also reported the distribution of foot (55.4% vs. 27.1%, p < 0.001) and thorax fractures (43.1% vs. 28.6%, p < 0.001) [5]. In our study, group III (intentional/not severe) comprised a lower proportion of patients with thorax fracture than group IV (accidental/not severe). Although no distinction was made between intentional and accidental falls, several studies that included only fatal cases reported that non-survivors had a higher proportion of head and thoracic injuries [11,12]. Regarding the distribution of trauma severity, Piazzalunga et al. reported the AIS scores for the head, face, thorax, abdomen, and extremities (upper and lower extremities) in intentional and accidental fall groups. They observed no significant difference in the AIS score for the head between the intentional and accidental fall groups (2.9 ± 1.25 vs. 2.59 ± 1.42) [4]. However, our study showed that the intentional fall group had higher AIS scores for region 1 (head, face, and neck) than the accidental fall group (Figure 2a). Such partially concordant and discordant findings resulted from the lack of comparisons adjusted for overall trauma severity such as ISS. In addition, these studies were single-institution case series, had small sample sizes, and did not have data on the distribution of trauma severity for each body region (AIS score).

Our results add to a comprehensive understanding of the differences between intentional and accidental falls from a height as our study resolved the abovementioned limitations. We used the JTDB which has more than 25,000 cases of falls from a height and summarized the distribution of trauma severity for each body region and overall trauma severity.

The difference between intentional and accidental falls from a height shown in our study can be attributed to the difference in body position when landing on the ground [4]. When patients land on their heels, the external force travels through the lower extremities and reaches the pelvis and vertebrae. In particular, unstable pelvic ring disruptions, such as vertical shear, are caused by high-energy blunt trauma including a fall from a height [13,14]. The skeletal trauma patterns in our results strongly support this finding. The difference in the ISS between the intentional and accidental fall groups was consistent with the difference in the AIS scores for the lower extremities and pelvis. Considering the ISS of the patients included in the previous studies, our results were consistent with the findings on skeletal trauma patterns reported in the previous studies.

This study also had some limitations. First, the JTDB is not a population-based sample of all trauma patients. Patients who were not registered in the participating hospitals or who were not transferred to hospitals due to on-scene death were not included in the JTDB; therefore, selection bias cannot be ruled out. However, our study included 3,581 patients who died in the ED, which would reduce the degree of bias to a certain extent. Second, the inaccuracies of the database itself may have influenced the results. Haas et al. highlighted underreporting of some items in the national trauma databank [15]. The JTDB would also have the same limitations. However, we only used variables that required no subjective assessment, except for AIS coding. The patients were, in principle, registered on the JTDB by medical assistants who had attended lectures on AIS coding. In addition, very few data were missing in our study. Therefore, the inaccuracy within the JTDB would not have significantly influenced our results.

## Conclusions

In this study, we demonstrated the differences in trauma patterns and trauma severity in patients who intentionally and accidentally fell from a height. Our findings are clinically relevant and can help emergency physicians to develop an initial treatment strategy for trauma patients injured by falls from a height at the ED. Further studies will be required to assess the impact of our results on the initial treatment strategy implemented at the ED.
